# Comparative efficacy of 5 suture configurations for arthroscopic rotator cuff tear repair: a network meta-analysis

**DOI:** 10.1186/s13018-021-02847-y

**Published:** 2021-12-11

**Authors:** Wei Wang, Hui Kang, Hongchuan Li, Jian Li, Yibin Meng, Peng Li

**Affiliations:** 1 Department of Shoulder and Elbow of Sports Medicine, Honghui Hospital, Xi’an Jiaotong University, Xian City, 710054 Shanxi Province China; 2 Departments of Spine Surgery, Honghui Hospital, Xi’an Jiaotong University, Xian City, 710054 Shanxi Province China; 3 Department of Hand Surgery, Honghui Hospital, Xi’an Jiaotong University, 76 Guo Road, Beilin South District, Xian City, 710054 Shanxi Province China

**Keywords:** Rotator cuff tears, Arthroscopy, Rotator cuff repair, Single row, Double row, Modified Mason–Allen, Suture bridge

## Abstract

**Background:**

Rotator cuff tear is one of the most common complaint with shoulder pain, disability, or dysfunction. So far, different arthroscopic techniques including single row (SR), double row (DR), modified Mason–Allen (MMA), suture bridge (SB) and transosseous (TO) have been identified to repair rotator cuff. However, no study has reported the comparative efficacy of these 5 suture configurations. The overall aim of this network meta-analysis was to analyze the clinical outcomes and healing rate with arthroscopy among SR, DR, MMA, SB and TO.

**Methods:**

A systematic literature was searched from PubMed, EBSCO-MEDLINE, Web of Science, google scholar and www.dayi100.com, and checked for the inclusion and exclusion standards. The network meta-analysis was conducted using Review Manager 5.3 and SATA 15.0 software.

**Results:**

Thirty-four studies were eligible for inclusion, including 15 randomized controlled trials, 17 retrospective and 2 prospective cohort studies, with total 3250 shoulders. Two individual reviewers evaluated the quality of the 34 studies, the score form 5 and 9 of 10 were attained according to the Newcastle–Ottawa Scale for the 17 retrospective and 2 prospective studies. There was no significant distinction for the Constant score among 5 groups in the 16 studies with 1381 shoulders. The treatment strategies were ranked as MMA, DR, SB, SR and TO. In ASES score, 14 studies included 1464 shoulders showed that no significant differences was showed among all 5 groups after surgery. Whereas the efficacy probability was TO, MMA, DR, SB and SR according to the cumulative ranking curve. The healing rate in 25 studies include 2023 shoulders was significant in both SR versus DR [risk ratio 0.45 with 95% credible interval (0.31, 0.65)], and SR versus SB [risk ratio 0.45 (95% credible interval 0.29, 0.69)], and no significant in the other comparison, the ranking probability was MMA, SB, DR, TO and SR.

**Conclusion:**

Based on the clinical results, this network meta-analysis revealed that these 5 suture configurations shows no significant difference. Meanwhile, suture bridge may be the optimum treatment strategy which may improve the healing rate postoperatively, whereas the DR is a suboptimal option for arthroscopic rotator cuff repairs.

## Introduction

Rotator cuff tear is a common problem that impairs the shoulder, and leads to the shoulder pain and poor function, along with insomnia [1]. The incidence of rotator cuff tear increases in people above 30 by 16–34% [2], and reaches approximately 54% in people in their 60s [3]. Only in the USA, the cost for treatment, evaluation, and management to this disease costs 3 billion US dollars, every year [4]. According to the. American Academy of Orthopaedic Surgeons reports that only 16% of rotator cuff tears had been managed and treated appropriately whereas 31% “may” have been appropriate, and 53% were “rarely appropriate” [5]. This situation was still a challenge for rotator cuff tear repair worldwide, with the need to promote functional recovery and increase the healing rate.

In recent years, significant development has been made in both the operative and conventional therapies of shoulder pain and pathological conditions [6]. The surgical remedy should be employed if conventional treatment fails. In arthroscopic rotator cuff repair, different suture techniques, with anchors, have been used worldwide, such as single row (SR), double row (DR), modified Mason–Allen (MMA), suture bridge (SB) and transosseous (SO). Even though development had been acquired recently with arthroscopic RCR by techniques of anchors fixation, the outcomes are not satisfactory. Therefore, the innovations in the methods for rotator cuff repair (RCR) are necessary. In clinical practice, surgeons had many choices based on personal experience, and the best treatment choice varies patient to patient. So far, no study has shown the comparative efficacy of different suture techniques, including SR, DR, MMA, SB and TO, used during arthroscopic RCR.

It is critical to evaluate the comparative efficacy, directly and indirectly, with the existing data using network meta-analysis, and summarize and explain the broader evidence to understand the advantages of different suture techniques. Our purpose was to prove which application in arthroscopic RCR would improve the shoulder function and tendon healing better. Therefore, this study aimed to perform the network meta-analysis for the currently available functional results and healing rate of arthroscopic RCR with SR, DR, MMA, SB and TO.

## Methods

### Search procedure

This network meta-analysis was conducted based on the Preferred Reporting Items for Systematic Reviews and Meta-Analysis guidelines. PubMed, EBSCO-MEDLINE, Web of Science, google scholar and www.dayi100.com were searched for articles published from January 2000 to March 2020 with the following words: “rotator cuff tears; arthroscopy; rotator cuff repair (RCR); single row (SR); double row (DR); modified Mason–Allen (MMA); suture bridge (SB); transosseous (TO)”. From PubMed, we used the search strategies “rotator cuff tears” AND “single row” OR “double row” OR “modified Mason–Allen” OR “suture bridge” OR “transosseous” assembled with all included literature.

### Inclusion criteria

The inclusion criteria included: (1) patients diagnosed with rotator cuff injury and repaired with arthroscopy; (2) the control group was any suture configurations of 5, they were compared between two groups respectively. (3) the studies included clinical functional outcomes and healing rate for all groups, with outcomes in accordance with Constant score system, the American shoulder or elbow surgeons score system (ASES). (4) clinical follow-up at least 6 months; (5) randomized controlled trial (RCT), prospective or retrospective cohort studies.

### Exclusion criteria

Case report, animal experiments, and basic medicine studies were excluded. Patients who underwent shoulder surgery were also excluded.

### Data extraction and quality assessment

The title and abstracts of all the searched literatures were accessed, the duplicates and animal trials were removed. Time of publication, study type, first author, patient information, surgical technique, clinical outcomes, and healing rate was listed into the standard form to compare. All of them were extracted by two independent authors (Peng Li and Hui Kang). Another author (Yibin Meng) crosschecked all the included and excluded studies for any discrepant opinion.

The quality of randomized controlled trials (RCT) was evaluated with Collaboration tool [7]. The judgment standard included six indexes: sequence generation, allocation hiding, blindness, incomplete result data, selective result reporting, and other “bias”, “low risk”, “high risk”, or “unclear” were the grading standard for each index of the included studies. According to the Newcastle–Ottawa Scale (NOS) [8], the quality of prospective and retrospective cohort studies was evaluated. The two authors (Hongchuan Li and Jian Li) independently assessed the quality of these literatures.

### Data analysis

Revman 5.3 software was employed for all conventional meta-analysis. The weighted mean difference (MD) and standard deviation (SD) were used to analyze the continuous variables (Constant, ASES), and relative risk was used to appraise the dichotomous variables (healing rate). Values were considered as statistically significant when *P* value < 0.05, including 95% CI. The *I*^2^ statistical was selected to test the heterogeneity of included studies (significance, *I*^2^ > 50%). For the pool outcomes of comparable studies, *I*^2^ > 50% was considered the significant heterogeneity and belong to the random-effect model. The network meta-analysis was based on a frequent framework with indirect and direct comparing. Stata software (version 15.0) was used to perform network, forest, and predictive interval plots [9, 10]. The rank of the five suture configurations for arthroscopic repair in the aspect of shoulder function and healing rate was assessed with the SUCRA [11]. The surface indicates the treatment efficacy, and the more surface shows, the better result. The inconsistencies were estimated with network side-split. The publication bias was judged with the funnel plot.

## Result

### Study identification and assessment

**Fig. 1:**
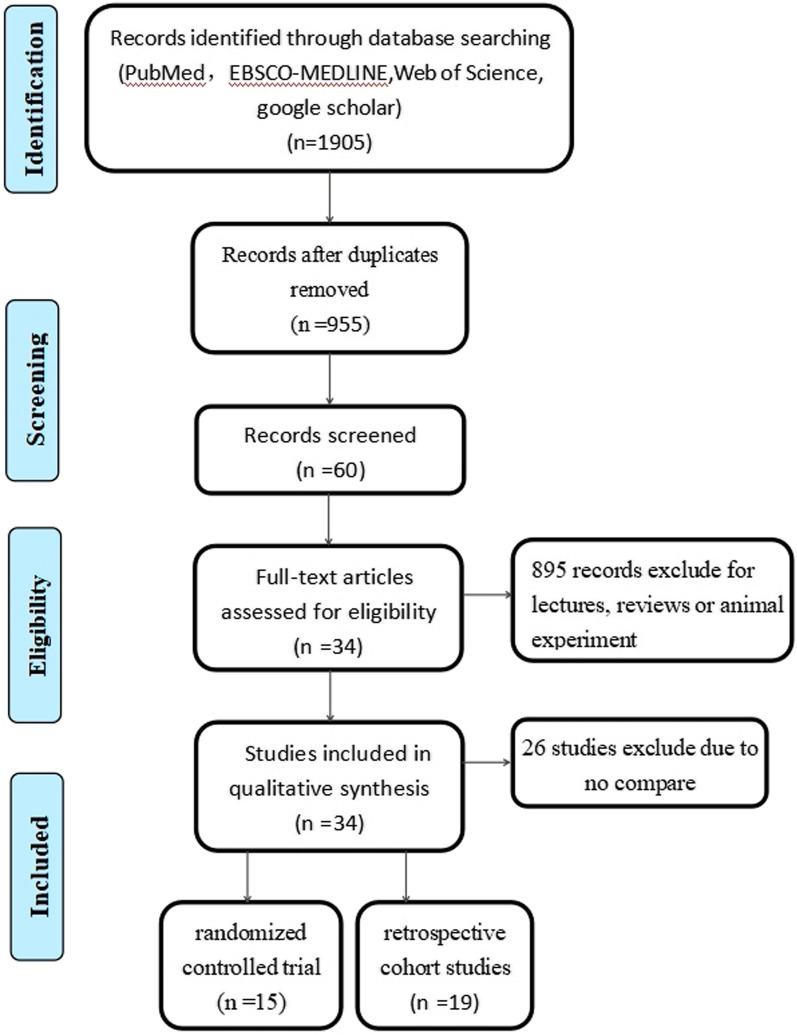
Search strategy flow diagram

One thousand ninety-five studies were identified, studies not fulfilling the inclusion criteria were excluded. Ultimately, 34 studies [12–45] fulfilled the inclusion criteria and the assessment in this network meta-analysis (Fig. 1). These studied include 15 RCTs [12, 17, 21, 22, 24, 25, 28, 30–34, 37, 41, 45], 2 prospective [16, 36] and 17 retrospective cohort studies [13–15, 18–20, 23, 26, 27, 29, 35, 38–40, 42–44], with a total of 3250 shoulders (Table 1).

**Table 1:** Basic data of included studies

First author	Year	Country	Evidence level	Interventions	Sample (shoulder)	Follow-up (months)	Study design	Outcome	Nos
Mohamed	2018	Egypt	III	MMA versus SB	21/25	49	RCT	Constant; ASES	–
Michael E	2018	Greece	II	SR versus SB	34/32	46	RCS	Constant; healing rate	7
Christian	2012	Germany	III	MMA versus SB	20/20	16	RCS	Constant; healing rate	8
Kwang	2018	Korea	III	MMA versus SB	39/37	35	RCS	Constant; ASES; healing rate	8
William	2010	USA	III	SR versus SB	78/54	24	PCS	ASES; healing rate	9
Atsushi	2017	Japan	II	TO versus SB	11/10	6	RCT	Constant	–
Teruhisa	2011	Japan	III	SR versus DR versus SB	65/23/107	24	RCS	ASES; healing rate	9
Cosimo	2013	Italy	III	SR versus DR	20/20	40	RCS	Constant; healing rate	8
Frank	2014	USA	III	SR versus DR versus SB	20/21/22	24	RCS	Constant; ASES; healing rate	9
Ignacio	2012	Spain	I	SR versus DR	80/80	24	RCT	Constant; ASES; healing rate	-
Gary M	2013	USA	I	SR versus SB	43/47	10	RCT	Healing rate	–
Ji-Sang	2019	Korea	III	SR versus SB	31/25	24	RCS	Constant; ASES; healing rate	7
Burks	2009	Australia	I	SR versus DR	20/20	12	RCT	ASES	–
Ma	2011	China	II	SR versus DR	27/20	24	RCT	Constant; ASES; healing rate	–
Charousset	2007	France	II	SR versus DR	35/31	28	RCS	ASES; healing rate	6
Park	2008	Korea	II	SR versus DR	40/38	24	RCS	Constant; healing rate	8
Franceschi	2007	Italy	I	SR versus DR	30/30	24	RCT	Constant; ASES	–
Sugaya	2005	Japan	III	SR versus DR	39/41	35	RCS	Healing rate	8
Kyoung	2011	Korea	I	SR versus DR	31/31	24	RCT	ASES; healing rate	–
Andrea	2009	Italy	I	SR versus DR	40/40	24	RCT	Constant; ASES; healing rate	–
Nuri	2009	Turkey	II	SR versus DR	34/34	24	RCT	Constant	–
Lapner	2012	Canada	I	SR versus DR	39/34	24	RCT	Constant	–
Francesco	2016	Italy	I	SR versus DR	25/25	24	RCT	Constant; ASES; healing rate	–
Eduard	2009	Switzerland	III	SR versus DR	32/33	25	RCS	Healing rate	8
Manuel	2020	Spain	II	SR versus SB	25/25	33	PCS	Constant	9
Randelli P	2017	California	I	TO versus SR	34/35	15	RCT	Constant; ASES; healing rate	–
Luís Filipe	2018	Brazil	III	SR versus DR	29/27	38	RCS	Constant; ASES; healing rate	8
Jeung	2017	Korea	III	SR versus SB	190/225	53	RCS	ASES	7
Jong-Hun	2010	Korea	III	SR versus DR	22/25	22	RCS	Constant; ASES; healing rate	8
Roshan	2017	India	II	SR versus DR	28/28	6	RCT	ASES; healing rate	–
Junji	2015	Japan	III	SR versus SB	25/36	81	RCS	Healing rate	7
Raffaele	2018	Italy	II	TO versus SR	54/42	24	RCS	Constant; ASES; healing rate	9
Robert Z	2018	USA	III	SR versus SB	22/25	12	RCS	ASES; healing rate	7
Francisco	2006	Spain	I	SR versus DR	50/50	26	RCT	Healing rate	–

*RCT* randomized controlled trial, *RCS* retrospective cohort study, *PCS* prospective cohort study, *SR* single-row, *DR* double-row, *SB* suture bridge, *MMA* modified Mason–Allen, *TO* transosseous

### Characteristics and quality assessments

**Fig. 2:**
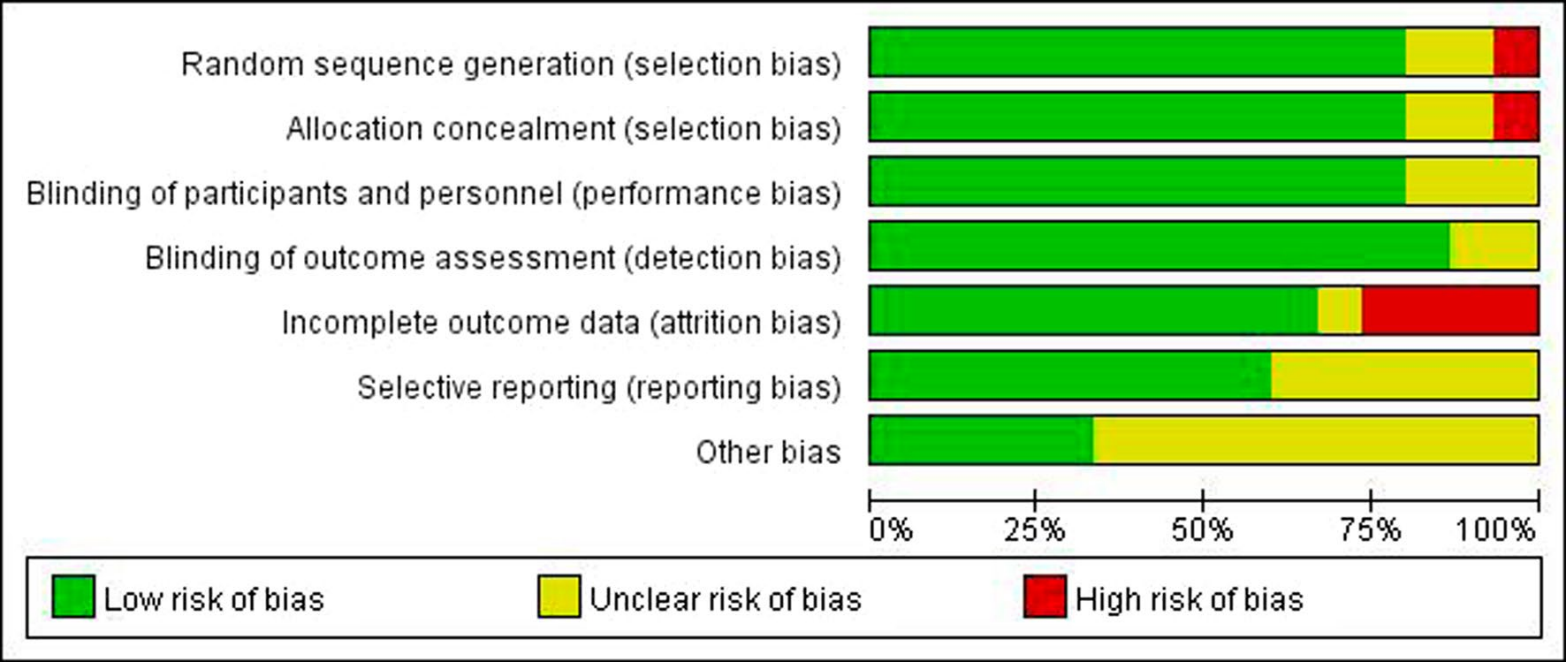
Risk of bias graph: review authors' judgements about each risk of bias item presented as percentages across all included RCTs

Table 1 shows the characteristics of the selected studies. The quality of 15 RCTs was assessed by two authors independently using the Cochrane Handbook for Systematic Reviews of Interventions 5.0 (Fig. 1). Furthermore, the NOS was applied to assess the pool bias of 2 prospective and 17 retrospective cohort studies (Fig. 2) to attain the score form 5 and 9 of 10.

### Constant score

**Fig. 3:**
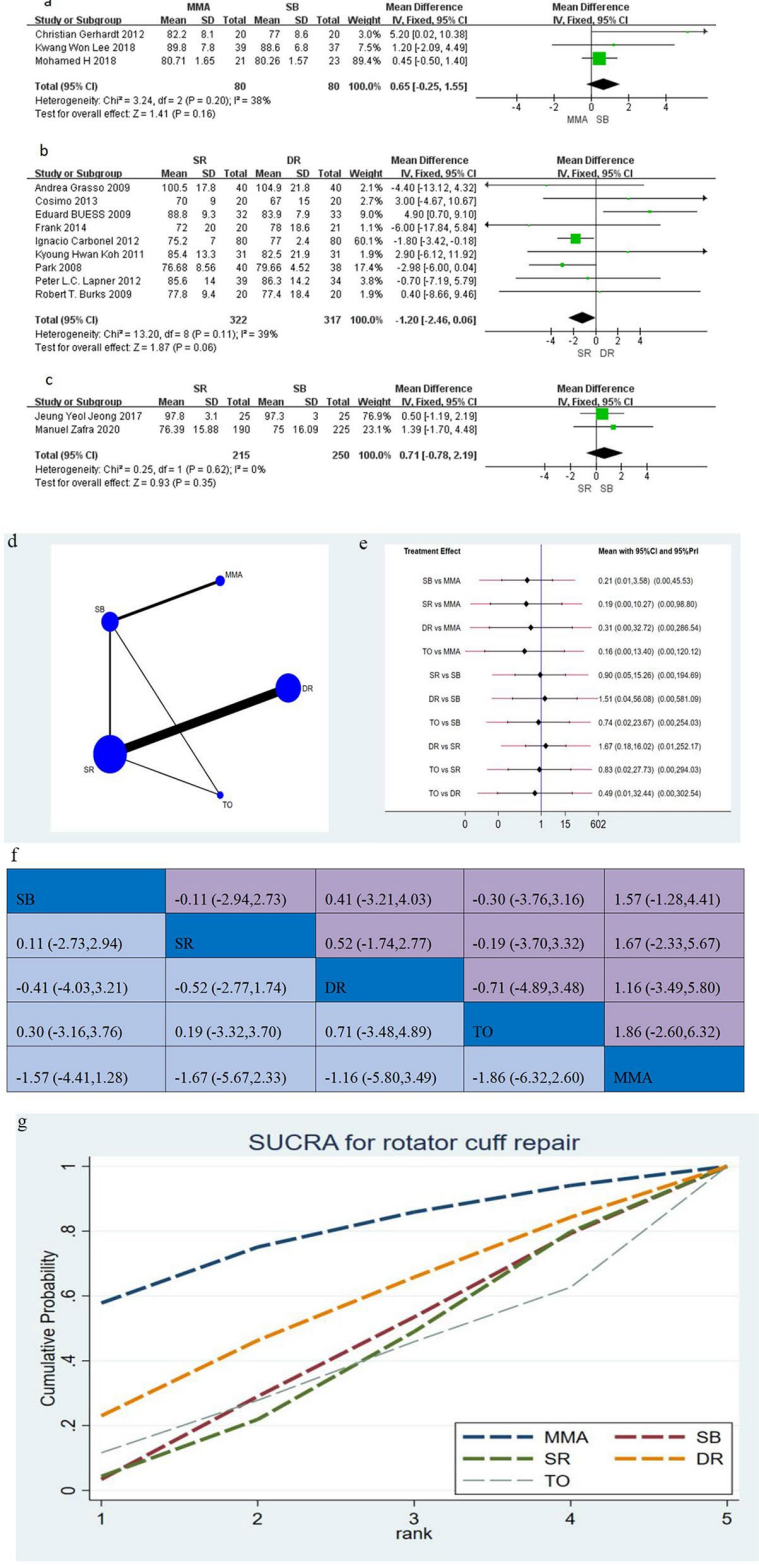
**a**–**c** The forest plot of conventional meta-analysis for Constant score; **d** network plot of suture configurations comparisons for Constant score. The size of the blue area indicates the sample size of each group, and the thickness indicates the studies of comparisons between two groups; **e** the predictive interval plot for Constant score; **f** head-to-head comparisons of network meta-analysis for Constant score; **g** the SURCA show the treatment efficacy of each suture configurations for Constant score. *MMA* modified Mason–Allen, *SB* suture bridge, *SR* single-row, *DR* double-row, *TO* transosseous

Sixteen studies [12, 14, 15, 17, 19–21, 24, 27, 30, 31, 33, 35, 36, 39, 43], including 1381 shoulders assess, the clinical functions using Constant score, showed postoperatively difference between two groups in this network meta-analysis. The conventional meta-analysis is presented in Fig. 3a–c (MD with 95% CI). The network plot between the five techniques, and the network meta-analysis is shown in Fig. 3d–g. In Constant score, direct and indirect comparison by conventional and network meta-analysis illustrated no significant differences among SR, DR, SB, MMA, and TO (Fig. 3e, f). According to the SUCRA (Fig. 3g), the ranking probability of the treatment efficacy of each suture configuration for Constant score was MMA, DR, SB, SR, and TO.

### ASES score

**Fig. 4:**
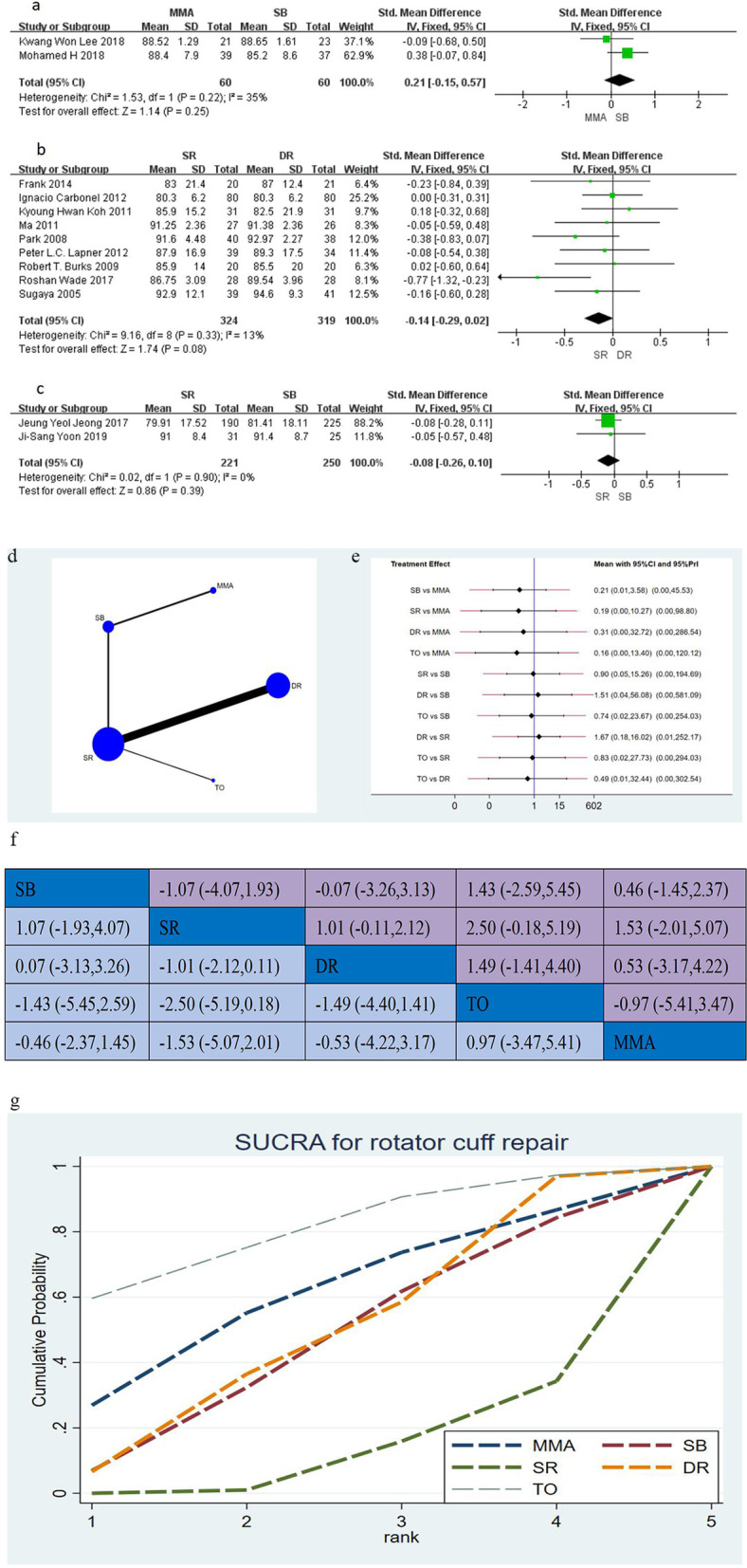
**a**–**c** The forest plot of conventional meta-analysis for ASES score; **d** network plot of suture configurations comparisons for ASES score. The size of the blue area indicates the sample size of each group, and the thickness indicates the studies of comparisons between two groups; **e** the predictive interval plot for ASES score; **f** head-to-head comparisons of network meta-analysis for ASES score; **g** the SURCA show the treatment efficacy of each suture configurations for ASES score. *MMA* modified Mason–Allen, *SB* suture bridge, *SR* single-row, *DR* double-row, *TO* transosseous

Regarding the ASES score, 14 studies [12, 15, 20, 21, 23–25, 27, 29, 30, 33, 39, 41, 43], including 1464 shoulders assess, the clinical function between the different two groups postoperatively in this network meta-analysis. The conventional meta-analysis is shown in Fig. 4a–c (MD with 95% CI). The network plot between the five techniques and network meta-analysis is shown in Fig. 4d–g. In the ASES score, it was no significant between any two sutures configurations in the 14 studies (Fig. 4e, f) with direct and indirect comparison by both conventional and network meta-analysis. On the basis of the SUCRA (Fig. 4g), the ranking probability of the treatment efficacy of each suture configuration for ASES score was TO, MMA, DR, SB and SR.

### Healing rate

**Fig. 5:**
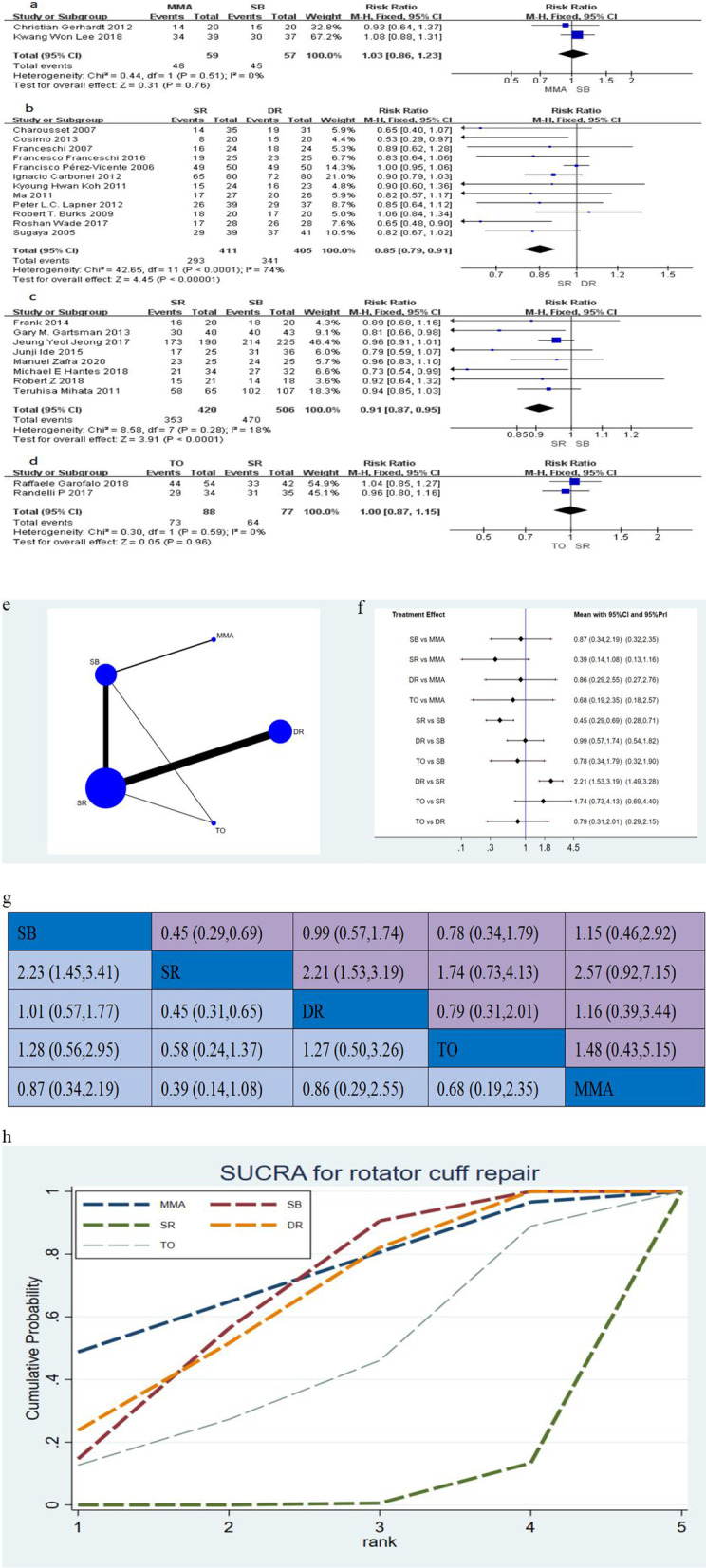
**a**–**d** The forest plot of conventional meta-analysis for healing rate; **e** network plot of suture configurations comparisons for healing rate. The size of the blue area indicates the sample size of each group, and the thickness indicates the studies of comparisons between two groups; **f** the predictive interval plot for healing rate; **g** head-to-head comparisons of network meta-analysis for healing rate; **h** the SURCA show the treatment efficacy of each suture configurations for healing rate. *MMA* modified Mason–Allen, *SB* suture bridge, *SR* single-row, *DR* double-row, *TO* transosseous

Twenty-five studies [13–16, 18–22, 24–26, 28–30, 33, 34, 36, 37, 39, 41–45], including 2023 shoulders assess, the healing rate between differentiate two groups postoperatively in this network meta-analysis. The conventional meta-analysis is presented in Fig. 5a–d (RR with 95% CI). The network plot between the 5 techniques and network meta-analysis is shown in Fig. 5e–h. Regarding the healing rate, there was significant differences both SR versus DR and SR versus SB in the network meta-analysis (Fig. 5g), and no significant in the other comparison (Fig. 5f, g). Judging from the SUCRA (Fig. 5h), the ranking probability of the treatment efficacy of each method for healing rate was MMA, SB, DR, TO and SR.

### Inconsistency and bias of publication analysis

**Fig. 6:**
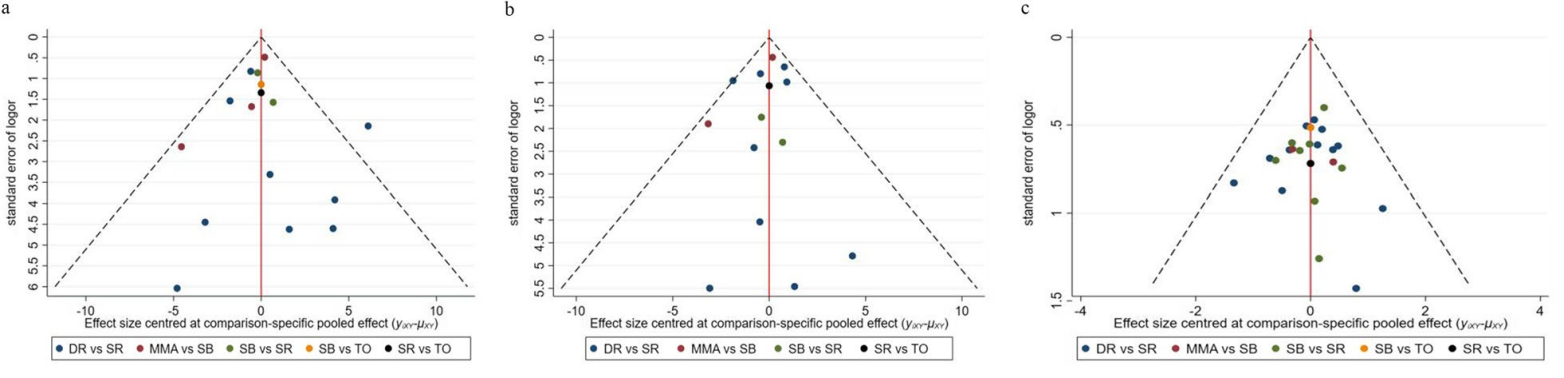
**a** The funnel plots of the included studies for constant score. **b** The funnel plots of the included studies for ASES score. **c** The funnel plots of the included studies for healing rate. *MMA* modified Mason–Allen, *SB* suture bridge, *SR* single-row, *DR* double-row, *TO* transosseous

The outcomes of pair-wise meta-analysis and the network meta-analysis matched significantly. In the study, there was no inconsistency for each result between the direct and indirect comparison (Table 2). Moreover, no visual evidence of bias of publication for each outcome was demonstrated from the funnel plots (Fig. 6), and it was similarly balanced on both sides of the funnel.

**Table 2:** Direct and indirect analysis for inconsistency of network meta-analysis

Outcome	Comparison	Direct		Indirect		Difference		*P* >|*z*|
		Coef.	Std.Err.	Coef.	Std.Err.	Coef.	Std.Err.	
Constant score	MMA versus SB	− 1.57	− 1.45	0.14	27.77	− 1.71	27.80	0.95
	SB versus SR	0.85	1.41	− 4.05	2.85	4.90	3.18	0.12
	SB versus TO	− 2.20	1.96	2.75	2.52	− 4.95	3.20	0.12
	SR versus DR	0.51	1.15	3.87	66.81	− 3.36	66.81	0.96
	SR versus TO	1.90	2.08	− 3.05	2.41	4.95	3.20	0.12
ASES sore	MMA versus SB	− 0.46	0.98	0.89	28.90	− 1.36	28.91	0.963
	SB versus SR	− 1.07	1.53	0.43	30.88	− 1.51	30.91	0.96
	SR versus DR	1.00	0.57	4.08	66.76	− 3.07	66.76	0.96
	SR versus TO	2.5	1.37	5.56	20.02	− 3.06	20.0	0.99
Healing rate	MMA versus SB	− 0.14	0.47	0.47	21.32	− 0.62	21.33	0.98
	SB versus SR	− 0.88	0.22	0.47	0.88	− 1.35	0.91	0.14
	SB versus TO	0.18	0.51	− 1.17	0.75	1.36	0.91	0.14
	SR versus DR	0.79	0.19	2.68	57.77	− 1.89	57.77	0.97
	SR versus TO	− 0.29	0.73	1.07	0.56	− 1.36	0.91	0.14

*MMA* modified Mason–Allen, *SB* suture bridge, *SR* single-row, *DR* double-row, *TO* transosseous

## Discussion

The network meta-analysis revealed the comparative efficacy of 5 suture configurations for rotator cuff tear in terms of the Constant score, ASES score, and healing rate for patients who underwent arthroscopic repair with MMA, SB, SR, DR, and TO. This study showed the following: (1) there was no significant differences among the five suture configurations in term of Constant score, and the overall ranking was MMA, DR, SB, SR, and TO; (2) there was no significant difference in ASES score, and the overall ranking was TO, MMA, DR, SB, and SR. (3) SR leading to a lower healing rate than DR and SB, and the ranking for healing rate was MMA, SB, DR, TO, and SR.

The constant score is a critical criterion for shoulder treatment including shoulder function, range of motion, pain and strength [46]. Gerhardt et al. [14] found that clinical results after MMA and SB techniques do not demonstrate significant differences in a matched patient cohort. Moreover, Hantes et al. [13] and his co-worker found no difference in Constant scores between the SR and DR techniques in a 46 months follow-up study having 66 patients. Furthermore, Garofalo et al. [43] reported that MMA repair provides comparable clinical results to SR repair in Constant score with arthroscopy. Zafra et al. [36] suggested that there were no differences in Constant score between SR and SB techniques. The network meta-analysis compared the difference among the 5 techniques combined direct and indirect evidences for rotator cuff repair with quantitative way, which illustrated no significant difference among 5 suture configurations. The SUCRA was used to assess the slight differences among MMA, SB, SR, DR and TO. In order to achieve better Constant score, the techniques can be arranged as follows: MMA, DR, SB, SR and TO.

The ASES score is essential for evaluating the therapeutic effect of these five arthroscopic techniques. No difference among the MMA, SB, SR, DR, and TO has been analyzed by the previous evidence-based study. Khalil et al. [12] previously reported that MMA provides comparable functional results to the SB repair technique. McCormick et al. [20] considered that using SR, DR, or SB techniques, yielded a clinical improvement and revealed no statistically significant difference for ASES score. Garofalo et al. [43] reported no statistically significant difference between SR and TO for the rotator cuff repair in the comparative analysis of ASES scores. No significant differences among MMA, SB, SR, DR and TO repair was found in terms of ASES score from this network meta-analysis. Furthermore, TO technique provided a greater ASES score than MMA, DR, SB and SR techniques according to the SUCRA.

As we all know that the critical point of RCT requires the repaired site tend-to-bone surface healing [47, 48]. One of the most common reasons for the failure of an RCR is the retear because of nonhealing of the primary repair [49]. A study by Park et al. [27] showed that approximately 50% of repaired rotator cuffs do not heal completely, though the surgical techniques were used. Hantes et al. [13] found that significant superior healing rate was potentially provided with the DR rather than the SR technique, which may due to the contact surface of tendon and bone. So Franceschi et al. [34] suggested that in selected patients with required accelerated postoperative rehabilitation, double-row repair lowered the risk of retear, while maintaining a low rate of stiffness. Tudisco et al. [19] reported that the healing rates after arthroscopic rotator cuff repair were 89.2% and 95.3%, respectively, for the SR and SB techniques, which was statistically significant. According to the SUCRA, the treatments efficacy was ranked as MMA, SB, DR, TO and SR repair based on their healing rate.

Our study has several advantages. Firstly, except for only direct groups compare, this network meta-analysis assesses five treatments simultaneously indirectly. As to our knowledge, this is the first time of comparison of MMA, SB, SR, DR, and TO techniques for arthroscopic rotator cuff repair. We compared the five different methods and supplied the SUCRA indirectly with a frequentist framework for network meta-analysis when no head-to-head compare existed by combining directly [50, 51]. Secondly, we avoided selection bias by synthesizing much more studies rather than a conventional meta-analysis. Additionally, this study could gain more precise effect assessments for the five techniques with an updated statistical approach of network meta-analysis.

However, there are also some limitations of the network meta-analysis: (1) some low-quality RCTs and two prospective and 17 retrospective cohort studies, which may impair the significance of the conclusions, but according to the NOS, the mostly score were more than 7. (2) The outcomes were incomplete in some included studies, imputed data were used in the analysis, as we used the same imputation method for the same treatment, the outcomes should still supply effective evaluation. (3) We did not perform a meta-analysis on tear size because the results were reported rarely in 2 studies. In our study, we compared the overall treatment efficacy for all types of rotator cuff repair. (4) Some potential publication biases in the study, it was similarly balanced on both sides of the funnel and demonstrated no visual evidence of publication bias for each result.

## Conclusion

Our network meta-analysis revealed that no significant difference was found for the functional outcomes among the five suture configurations. SB repairs might be the optimum treatment and improve the healing rate postoperatively. Meanwhile, the DR is a suboptimal option for arthroscopic rotator cuff repairs, which may help and guide clinicians on the appropriate operative program.

## Data Availability

All data generated or analyzed during this study are included in this published article.
